# Neuroprotective strategies in acute aortic dissection: an analysis of the UK National Adult Cardiac Surgical Audit

**DOI:** 10.1093/ejcts/ezab192

**Published:** 2021-05-08

**Authors:** Umberto Benedetto, Arnaldo Dimagli, Graham Cooper, Rakesh Uppal, Giovanni Mariscalco, George Krasopoulos, Andrew Goodwin, Uday Trivedi, Simon Kendall, Shubhra Sinha, Daniel Fudulu, Gianni D Angelini, Geoffrey Tsang, Enoch Akowuah, the UK Aortic Surgery

**Affiliations:** 1 Bristol Heart Institute, University of Bristol, Bristol, UK; 2 Sheffield Teaching Hospitals Foundation Trust, Sheffield, UK; 3 Barts Heart Centre, William Harvey Research Institute, London, UK; 4 Department of Cardiac Surgery, Glenfield Hospital, Leicester, UK; 5 Oxford University Hospitals NHS Foundation Trust, Oxford, UK; 6 South Tees Hospitals NHS Trust, Middlesbrough, UK; 7 Sussex Cardiac Center, Brighton and Sussex University Hospitals NHS Trust, Brighton, UK; 8 Wessex Cardiothoracic Center, University Hospital Southampton NHS Trust, Southampton, UK

**Keywords:** Type A aortic dissection, Neuroprotection, Antegrade cerebral perfusion, Deep hypothermia, Cerebrovascular accidents

## Abstract

**OBJECTIVES:**

The risk of brain injury following surgery for type A aortic dissection (TAAD) remains substantial and no consensus has still been reached on which neuroprotective technique should be preferred. We aimed to investigate the association between neuroprotective strategies and clinical outcomes following TAAD repair.

**METHODS:**

Using the UK National Adult Cardiac Surgical Audit, we identified 1929 patients undergoing surgery for TAAD (2011–2018). Deep hypothermic circulatory arrest (DHCA) only, unilateral (uACP), bilateral antegrade cerebral perfusion (bACP) and retrograde cerebral perfusion were used in 830, 117, 760 and 222 patients, respectively. The primary end point was a composite of death and/or cerebrovascular accident (CVA). Generalized linear mixed model was used to adjust the effect of neuroprotective strategies for other confounders.

**RESULTS:**

The use of bACP was associated with longer circulatory arrest (CA) compared to other strategies. There was a trend towards lower incidence of death and/or CVA using uACP only for shorter CA. In particular, primary end point rate was 27.7% overall and 26.5%, 12.5%, 28.0% and 22.9% for CA <30 min and 28.6%, 30.4%, 33.3% and 33.0% for CA ≥30 min with DHCA only, uACP, bACP and retrograde cerebral perfusion, respectively. The use of DHCA only was associated with five-fold [odds ratio (OR) 5.35, 95% confidence interval (CI) 1.36–21.02] and two-fold (OR 1.77, 95% CI 1.01–3.09) increased risk of death and/or CVA compared to uACP and bACP, respectively, but the effect of uACP was significantly associated with CA duration (hazard ratio 0.97, 95% CI 0.94–0.99; *P* = 0.04).

**CONCLUSIONS:**

In TAAD repair, the use of uACP and bACP was associated with a lower adjusted risk of death and/or CVA when compared to DHCA. uACP can offer some advantage but only for a shorter CA duration.

**Figure:**
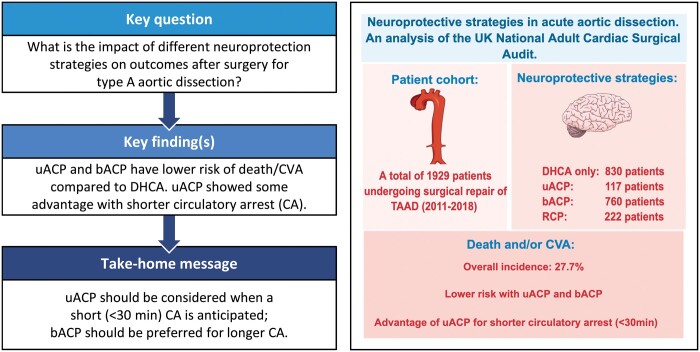

## INTRODUCTION

Management of type A acute aortic dissection (TAAD) remains a challenge. In many cases, surgical repair requires a period of circulatory arrest (CA) which continues to be associated with high rates of neurological complications and reduced survival [1–3] and subsequent increased length of intensive care unit stay and length of hospitalization [4]. Therefore, the need to optimize neuroprotection strategies during these operations is substantial, and defining optimal protective strategies remains an active area of research and debate within the cardiothoracic surgical community. The exclusive use of deep hypothermic CA (DHCA) has long been considered the standard neuroprotection strategy during surgery for TAAD [5, 6]. However, this technique remains associated with significant risk of brain injury [7].

In order to reduce the risk of brain injury, continuous cerebral perfusion techniques have been proposed including retrograde cerebral perfusion (RCP) via the venous system and antegrade cerebral perfusion (ACP) via the right subclavian artery only (unilateral ACP, uACP) or with selective perfusion of both the carotid arteries (bilateral ACP, bACP) [8–10]. The European Society of Cardiology guidelines on aortic disease recommend the adoption of selective ACP to reduce the risk of stroke for surgical aortic procedures (class IIa, level B) [11]. However, this recommendation mainly relies on evidence from observational studies with only few randomized clinical trials available limited by small sample size [3]. The final consensus on which neuroprotective strategy should be preferred is still lacking, and significant variation in the clinical practice exists [8–10].

Hence, we analysed a national dataset to get further insights into the association between different neuroprotective strategies and clinical outcomes following surgery for TAAD.

## METHODS

The study was approved by Health Research Authority (HRA) and Health and Care Research Wales (HCRW), and a waiver for patients’ consent was obtained (IRAS ID: 278171).

The National Institute for Cardiovascular Outcomes Research (NICOR) National Adult Cardiac Surgery Audit (NACSA) registry was interrogated for patients who underwent adult cardiac surgery from 2011 to 2018. This registry prospectively collects demographic, as well as preoperative, perioperative and postoperative clinical information and mortality information for all major adult cardiac surgery procedures performed in the UK, and its key function is benchmarking surgical practice.

This dataset undergoes regular robust validation and checking procedures with the aim to achieve and maintain high-quality standards of the information within it [12, 13]. There is an established policy for the handling of missing data. Missing and conflicting data for in-hospital mortality status are backfilled and validated via record linkage to the Office for National Statistics (ONS) census database*.* In case of missing data in variables required to calculate the EuroSCORE, it is assumed that the risk factor is not present, i.e. equivalent to the reference level. For any record of missing patient age at the time of surgery, the median patient age for the corresponding financial year is imputed. The overall percentage of missing data in the EuroSCORE risk factors was very low (1.7%) and missing records of patient age was <0.003%. No missingness was present for data regarding the neuroprotection strategy adopted in each patient and the primary end points.

From the NACSA registry, we identified patients undergoing surgery for TAAD requiring circulatory with information available neuroprotective strategies adopted. We defined four groups of patients on the basis of the neuroprotective strategy adopted: (i) use of DHCA only, (ii) use of uACP (with or without concomitant DHCA), (iii) use of bACP (with or without combined DHCA) and (iv) use RCP perfusion. Only a few patients (*n* = 20) received combined ACP and RCP and they were excluded from the analysis.

The primary outcome was a composite of in-hospital mortality and/or new postoperative cerebrovascular accident (CVA). In-hospital mortality and new postoperative CVA were also reported as isolated secondary end points along with the need for postoperative dialysis and re-exploration for bleeding.

### Statistical analysis

Categorical variables were summarized as counts and percentages. Continuous variables were summarized as mean and standard deviation (SD). Confounders considered included variables related to clinical presentation (i.e. age, gender, known Marfan disease, critical preoperative state, emergency surgery, pre-existent neurological deficit, diabetes, chronic pulmonary disease, extracardiac arteriopathy, previous cardiac surgery and creatinine >200 mmol/l) and procedural details (i.e. concomitant aortic valve surgery, full root replacement, coronary artery bypass graft surgery, arch debranching, concomitant endovascular procedure, total CA and total cross-clamp time). Outcomes of interest were presented as crude incidences across the four neuroprotective strategy groups. The primary end point was further stratified by duration of CA using a cut-off of 30 min chosen on the basis of previous reports [14, 15] and by the concomitant use of deep hypothermia (DH) for cerebral perfusion strategies. Finally, baseline characteristics and outcomes were also presented in subgroups of patients with similar extension of the surgical repair (i.e. aortic root replacement, arch replacement or ascending aorta only).

As neuroprotective strategy groups were different for baseline characteristics and intraoperative data (i.e. duration of CA), a multivariable generalized linear mixed model was used to estimate the effect of different strategies on the primary end point by accounting for other confounders. Individual hospitals, surgeons and year of surgery were included in the model as a random effect (random intercepts). The association between significant random effects and the adjusted risk of the primary end point was reported as variance (*σ*^2^). To test the interaction between different neuroprotective strategies and CA duration, an interaction term was forced in the model. Non-linearity for continuous variables was tested using spline and polynomial terms. Generalized variance inflation factor was used to test collinearity and variance inflation factors exceeding 10 were considered signs of serious multicollinearity. The marginal *R*-squared considers only the variance of the fixed effects, while the conditional *R*-squared takes both the fixed and random effects into account [16]. Effect estimates for fixed terms were reported as odds ratio (OR) and relative 95% confidence interval (CI). *P*-value <0.05 was considered significant in all the analysis. There was no pre-specified plan to adjust for multiple comparisons, and therefore *P*-values may not be interpreted as confirmatory but rather descriptive and inferences drawn from them may not be reproducible. All analyses were performed in R version 4.0.0. lme4, lmerTest and sjPlot packages were used to fit and present generalized linear mixed model results.

## RESULTS

A total of 1929 patients from 36 cardiac units who underwent TAAD repair with CA were included in the present analysis. The most commonly adopted neuroprotective strategy was DHCA only, used in 830 patients. uACP was adopted in 117 patients (concomitant DH was used in 69, 59%). bACP was used in 760 patients (concomitant DH was used in 482, 63.4%). RCP was adopted in 222 patients (concomitant DH was used in 161, 72.5%).

**Figure 1:**
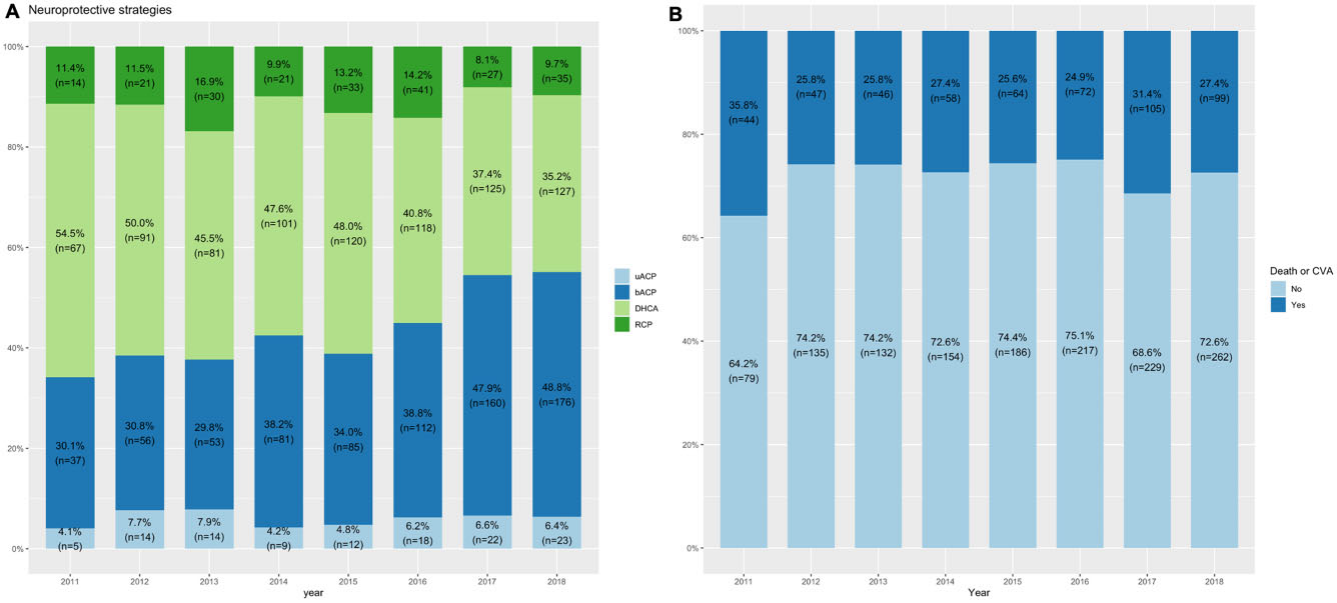
Prevalence of neuroprotective strategies adopted (**A**) and incidence of the primary end point (**B**) across years. bACP: bilateral antegrade cerebral perfusion; CVA: cerebrovascular accident; DHCA: deep hypothermic circulatory arrest; RCP: retrograde cerebral perfusion; uACP: unilateral antegrade cerebral perfusion.

The adoption of different neuroprotective strategies varied over the time with an increased use of bACP at the expense of DHCA only (Fig. 1A). Baseline characteristics and operative variables across the four groups and in the overall sample are reported in Table 1. The four groups differed for baseline characteristics (i.e. presence of a critical preoperative state) and surgical characteristics (i.e. extent of surgical repair and concomitant procedures). In particular, bACP group tended to have a higher risk profile and a higher proportion of patients requiring arch replacement and was associated with a longer CA and cross-clamp time.

**Table 1:** Baseline characteristics of patients in the four treatment groups

	DHCA (*N* = 830)	uACP (*N* = 117)	bACP (*N* = 760)	RCP (*N* = 222)	Overall (*N* = 1929)
Age (years),					
mean (SD)	61.9 (13.2)	62.3 (15.4)	62.5 (13.6)	63.4 (13.5)	62.3 (13.5)
Female gender, *n* (%)	292 (35.2)	40 (34.2)	254 (33.4)	78 (35.1)	664 (34.4)
Known Marfan, *n* (%)	28 (3.4)	4 (3.4)	24 (3.2)	9 (4.1)	65 (3.4)
Critical preoperative state, *n* (%)	42 (5.1)	6 (5.1)	63 (8.3)	30 (13.5)	141 (7.3)
Emergency, *n* (%)	712 (85.8)	97 (82.9)	591 (77.8)	197 (88.7)	1597 (82.8)
Neurological deficit, *n* (%)	52 (6.3)	10 (8.5)	76 (10.0)	22 (9.9)	160 (8.3)
Diabetes, *n* (%)	29 (3.5)	2 (1.7)	26 (3.4)	11 (5.0)	68 (3.5)
Chronic pulmonary disease, *n* (%)	64 (7.7)	10 (8.5)	58 (7.6)	18 (8.1)	150 (7.8)
Extracardiac arteriopathy, *n* (%)	135 (16.3)	27 (23.1)	207 (27.2)	30 (13.5)	399 (20.7)
Previous cardiac surgery, *n* (%)	47 (5.7)	6 (5.1)	58 (7.6)	7 (3.2)	118 (6.1)
Creatinine >200 mmol/l, *n* (%)	29 (3.5)	4 (3.4)	23 (3.0)	17 (7.7)	73 (3.8)
Aortic valve surgery, *n* (%)	329 (39.6)	34 (29.1)	249 (32.8)	71 (32.0)	683 (35.4)
Repair extension, *n* (%)					
Aortic root replacement	215 (25.9)	26 (22.2)	157 (20.7)	46 (20.7)	444 (23.0)
Aortic arch replacement	11 (1.3)	7 (6.0)	49 (6.4)	4 (1.8)	71 (3.7)
Root and arch replacement	5 (0.6)	1 (0.9)	14 (1.8)	1 (0.5)	21 (1.1)
Ascending aorta only	599 (72.2)	83 (70.9)	540 (71.1)	171 (77.0)	1393 (72.2)
CABG, *n* (%)	109 (13.1)	8 (6.8)	91 (12.0)	20 (9.0)	228 (11.8)
Cumulative cross-clamp time (min), mean (SD)	126 (69.1)	120 (53.0)	141 (70.4)	112 (53.9)	130 (67.9)
Circulatory arrest time, mean (SD)	33.4 (22.2)	34.7 (20.5)	43.7 (36.0)	31.2 (18.5)	37.2 (28.5)
DH, *n* (%)	830 (100)	69 (59.0)	482 (63.4)	161 (72.5)	1542 (79.9)

bACP: bilateral antegrade cerebral perfusion; CABG: coronary artery bypass grafting; DH: deep hypothermia; DHCA: deep hypothermic circulatory arrest; RCP: retrograde cerebral perfusion; SD: standard deviation; uACP: unilateral antegrade cerebral perfusion.

Overall, mean cross-clamp time and CA time were 130 (SD 67.9) and 37.2 (SD 28.5) min, with 50% and 75% of patients requiring a CA <30 and <45 min, respectively (Supplementary Material, Fig. S1).

**Figure 2:**
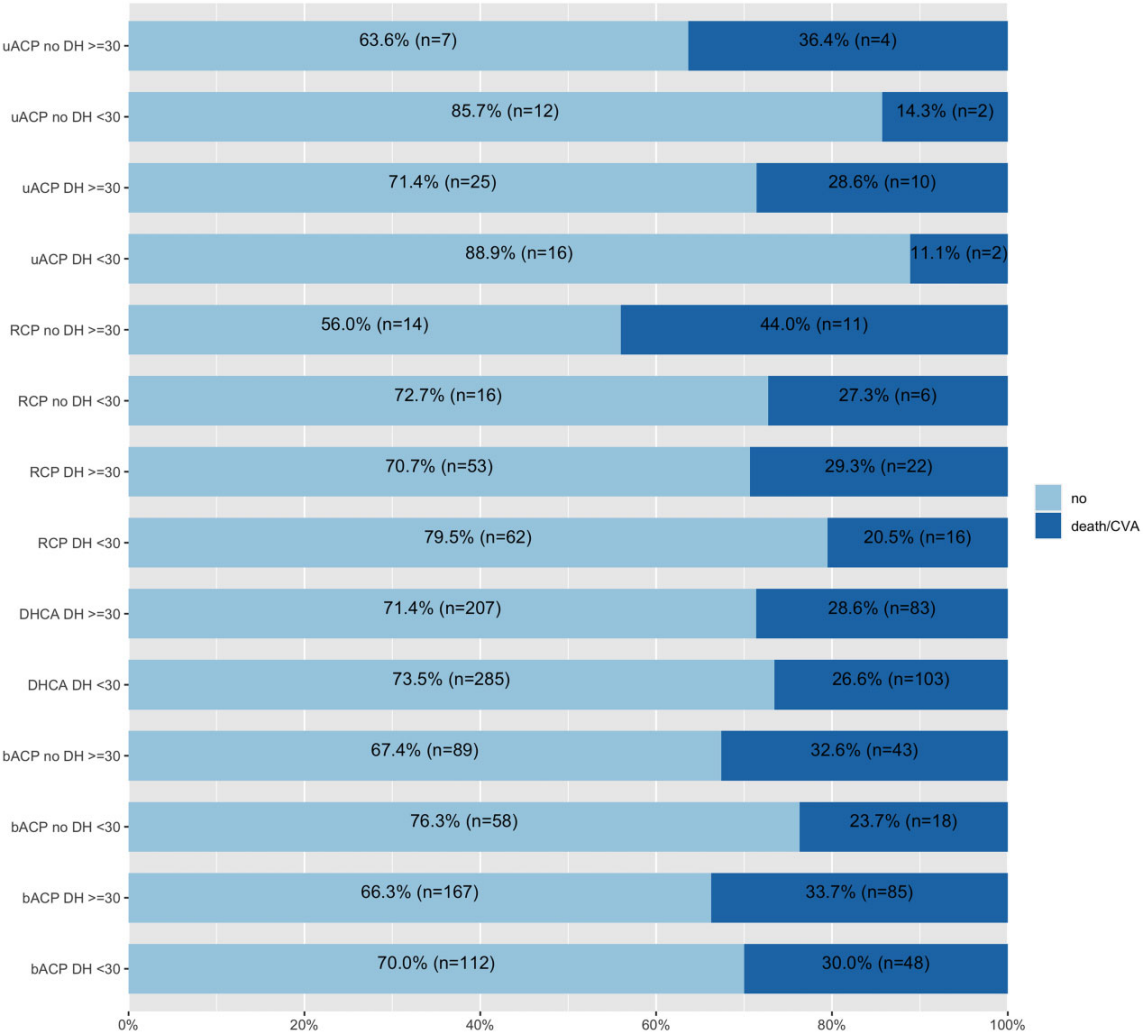
Incidence of adverse event (composite of death and CVA) across neuroprotective strategy groups stratified by duration of circulatory arrest and concomitant use of DH. bACP: bilateral antegrade cerebral perfusion; CVA: cerebrovascular accident; DH: deep hypothermia; RCP: retrograde cerebral perfusion; uACP: unilateral antegrade cerebral perfusion.

The overall incidence of the composite of death or CVA, mortality, CVA, dialysis and re-exploration was 27.7%, 17.4%, 13.9%, 14.1% and 10.4%, respectively. The incidence of the primary end point did not significantly vary over the time (Fig. 1B). Crude incidences for the primary and secondary end points in each neuroprotection group are reported in Table 2. The incidence of death and/or CVA was numerically lower in the uACP group. When the analysis was stratified by CA time duration, the advantage from uACP was evident only for CA time <30 min but not >30 min. In particular, primary end point rate was 26.5%, 12.5%, 28.0% and 22.9% for CA <30 min and 28.6%, 30.4%, 33.3% and 33.0% for CA ≥30 min with DHCA only, uACP, bACP and RCP, respectively (Supplementary Material, Table S1). This trend was also confirmed when we further stratified for concomitant use of DH (Supplementary Material, Tables S2 and S3 and Fig. 2).

**Table 2:** Incidence of primary and secondary end points in the four treatment groups

	DHCA (*N* = 830), *n* (%)	uACP (*N* = 117), *n* (%)	bACP (*N* = 760), *n* (%)	RCP (*N* = 222), *n* (%)	Overall (*N* = 1929), *n* (%)
Death or CVA					
No	606 (73.0)	91 (77.8)	537 (70.7)	160 (72.1)	1394 (72.3)
Yes	224 (27.0)	26 (22.2)	223 (29.3)	62 (27.9)	535 (27.7)
Death					
No	692 (83.4)	98 (83.8)	625 (82.2)	179 (80.6)	1594 (82.6)
Yes	138 (16.6)	19 (16.2)	135 (17.8)	43 (19.4)	335 (17.4)
CVA					
No	712 (85.8)	106 (90.6)	649 (85.4)	193 (86.9)	1660 (86.1)
Yes	118 (14.2)	11 (9.4)	111 (14.6)	29 (13.1)	269 (13.9)
Postoperative dialysis					
No	734 (88.4)	106 (90.6)	629 (82.8)	188 (84.7)	1657 (85.9)
Yes	96 (11.6)	11 (9.4)	131 (17.2)	34 (15.3)	272 (14.1)
Re-exploration					
No	752 (90.6)	107 (91.5)	674 (88.7)	195 (87.8)	1728 (89.6)
Yes	78 (9.4)	10 (8.5)	86 (11.3)	27 (12.2)	201 (10.4)

bACP: bilateral antegrade cerebral perfusion; CVA: cerebrovascular accident; DHCA: deep hypothermic circulatory arrest; RCP: retrograde cerebral perfusion; uACP: unilateral antegrade cerebral perfusion.

When the analysis for the primary end point was adjusted for confounders (Table 3 and Supplementary Material, Table S4), the use of DHCA only was associated with five-fold (OR 5.35, 95% CI 1.36–21.02; *P* = 0.016) and two-fold (OR 1.77, 95% CI 1.01–3.09; *P* = 0.045) increased risk of death or CVA when compared to uACP and bACP, respectively, while no significant difference was observed with RCP (OR 1.92, 95% CI 0.77–4.79; *P* = 0.163). There was a significant interaction between CA duration and the use of uACP (interaction OR 0.97, 95% CI 0.94–1.00; *P* = 0.039), while no interaction was found for bACP (interaction OR 0.99, 95% CI 0.98–1.00; *P* = 0.122) and RCP (interaction OR 0.99, 95% CI 0.97–1.01; *P* = 0.235). A significant effect of individual hospitals on the adjusted risk of the composite end point (*σ*^2^ = 0.37; *P* < 0.001) was found.

**Table 3:** Generalized mixed linear model results using unilateral antegrade cerebral perfusion as reference

	Composite (death/CVA)		
Predictors	Odds ratios	CI	*P*-value
Intercept	0.00	0.00–0.01	**<0.001**
Age (increase per year)	1.03	1.02–1.04	**<0.001**
Female gender	0.91	0.71–1.15	0.423
Known Marfan	0.38	0.15–0.96	**0.040**
Critical preoperative state	2.88	1.92–4.32	**<0.001**
Emergency	2.83	1.98–4.06	**<0.001**
Neurological dysfunction	1.75	1.19–2.57	**0.004**
Diabetes	0.59	0.31–1.11	0.103
Chronic pulmonary disease	2.03	1.37–2.99	**<0.001**
Extracardiac arteriopathy	1.11	0.83–1.49	0.492
Previous cardiac surgery	2.43	1.49–3.95	**<0.001**
Creatinine > 200 mmol/l	1.17	0.67–2.04	0.589
Aortic valve surgery	1.00	0.76–1.31	0.996
Full root replacement	1.01	0.73–1.41	0.930
CABG	2.26	1.61–3.17	**<0.001**
Arch debranching	2.16	0.97–4.78	0.058
Endovascular procedure	1.69	0.89–3.23	0.111
DHCA versus uACP	5.35	1.36–21.02	0.016
DHCA versus bACP	1.77	1.01–3.09	**0.04**
DHCA versus RCP	1.92	0.77–4.79	0.163
Circulatory arrest time	1.04	1.01–1.07	**0.008**
Interaction with circulatory arrest time			
DHCA versus bACP	0.98	0.95–1.01	**0.135**
DHCA versus uACP	0.97	0.94–1.00	**0.039**
DHCA versus RCP	0.98	0.95–1.02	0.271
Random effects			
σ^2^	3.29		
τ_00_ _hospital_	0.37		
ICC	0.10		
*N* _hospital_	36		
Observations	1851		
Marginal *R*^2^/Conditional *R*^2^	0.172/0.256		

bACP: bilateral antegrade cerebral perfusion; CABG: coronary artery bypass grafting; CI: confidence interval; CVA: cerebrovascular accident; DHCA: deep hypothermic circulatory arrest time; ICC: interclass correlation coefficient; RCP: retrograde cerebral perfusion; uACP: unilateral antegrade cerebral perfusion.

When the analysis was stratified according to the extension of the surgical repair (Supplementary Material, Tables S5 and S6), we found that bACP was largely preferred when the arch replacement was performed. Only 8 and 9 patients requiring arch replacement received uACP and RCP, respectively. As consequence, no conclusion on the impact of uACP and RCP in this complex setting could be derived.

## DISCUSSION

Postoperative cerebrovascular complications remain a major challenge in the treatment of TAAD, particularly in those cases requiring CA. Cerebral protection strategy has been an important focus to improve clinical outcomes in this high-risk setting. Historically, TAAD has been treated with DHCA only, introduced by Griepp *et al.* in 1975 [5]. This strategy is based on the depression of cerebral and systemic metabolism through DH. However, outcomes following TAAD repair using DHCA only remain suboptimal especially in case of prolonged CA time [7]. Although cerebral perfusion strategies (both ACP and RCP) with or without concomitant adoption of DH have become a more favoured approach among the contemporary aortic surgeons, there is still no universal consensus on which should be considered the default strategy [8–10]. Ethical and sample size constrain in these high-risk group of patients is possibly one of the reasons why a randomized trial comparing different cerebral perfusion strategies has yet to be performed. Therefore, our clinical practice relies on the best evidence from observational studies. Most of the previous studies are limited by a small sample, single-centre experience and they have focused mainly on the comparison between bilateral and unilateral ACP [8–10]. A summary of recent studies comparing the different neuroprotective strategies in TAAD surgical repair is presented in Table 4 [14, 15, 17–19].

**Table 4:** Summary of recent studies reporting on the outcomes of different neuroprotective strategies in TAAD surgical repair

Author (year)	Number of patients	Patient groups	Outcomes	Authors’ conclusions
Preventza *et al.* (2014) [14]	157	uACP: 90	Mortality: 13.3%	uACP may provide valuable technical simplicity and bACP may be justified for CA >30 min
			CVA: 14.8%	
		bACP: 63	Mortality: 12.7%	
			CVA: 12.9%	
Tobias *et al.* (2011) [15]	1558	DHCA only: 355	Mortality: 19.4%	CA <30 min: DHCA and ACP have similar results CA >30 min: ACP with sufficient pressure is advisable Outcomes with unilateral and bACP were equivalent
			CVA: 14.9%	
		uACP: 628	Mortality: 15.9%	
			CVA: 14.1%	
		bACP: 453	Mortality: 13.9%	
			CVA: 12.6%	
Norton *et al.* (2020) [17]	307	uACP: 140	uACP: bACP odds ratio CVA = 0.87 (*P* = 0.80) Mortality = 0.86 (*P* = 0.81)	Both strategies were equally effective
		bACP: 167		
Tong *et al.* (2017) [18]	203	uACP: 82	30-Day mortality: 20.7%	No significant difference in outcome between uACP and bACP
			CVA: 16.9%	
		bACP: 121	30-Day mortality: 11.6%	
			CVA: 8.4%	
Lu *et al.* (2012) [19]	263	uACP + DHCA: 135	CVA: 10.4%	uACP is safe and non-inferior to bACP
		bACP + DHCA: 128	CVA: 12.5%	

ACP: antegrade cerebral perfusion; bACP: bilateral antegrade cerebral perfusion; CA: circulatory arrest; CVA: cerebrovascular accident; DHCA: deep hypothermic circulatory arrest; TAAD: type A aortic dissection; uACP: unilateral antegrade cerebral perfusion.

To the best of our knowledge, the present report is the largest nationwide available series comparing all four neuroprotective strategies during CA for TAAD repair. The incidence of death or CVA was consistent with previous reports. We found evidence of a potential advantage from uACP in case of the short period of CA (<30 min), while this advantage was no longer present for a prolonged period of CA. Notably, concomitant DH was used in more than half of patients treated with uACP and this may have contributed to the benefit observed with uACP. Moreover, uACP was rarely adopted in patients requiring arch replacement while bACP was largely preferred in this more complex setting and this may have influenced the observed outcomes. After accounting for major confounders, both uACP and bACP were found superior to the use of DHCA only but the effect of uACP was dependent on duration of CA.

A possible benefit by preferring uACP over bACP for shorter period of CA can be partially explained by the increased risk of antegrade embolism (both gaseous and solid) and intimal damage to the carotid artery vessels due to direct manipulation with bACP [8]. This drawback of bACP could counteract the advantage of maintaining brain perfusion during CA. The use of uACP via right subclavian artery can minimize these risks while maintaining an adequate cerebral flow. On the other hand, the potential shortcoming of unilateral brain perfusion is likely to have a detrimental effect only for prolonged period of CA and particularly in patients who have an incomplete circle of Willis [20]. This could explain the increase in the risk of adverse events beyond the safety limit of 30 min.

Notably, in the present series, the incidence of death or CVA did not significantly improve over a period of 10 years despite bACP has progressively become the most common strategy (from 31% to 49%) at the expense of DHCA only (from 54% to 35%). On the other hand, the proportion of patients treated with uACP was small (∼6%) and did not increase over time. The low adoption of uACP could be explained by the common perception of increased vulnerability to brain injury with unilateral cerebral perfusion.

Finally, we found a strong effect of individual hospitals on outcome variation. This highlights the need for local and national quality improvement programs and suggests that clinical outcomes could be significantly improved by establishing a network of specialized centres for the treatment of patients with TAAD [21].

### Limitations

The present study suffers several limitations including the non-randomized design. It is possible that the increased advantage seen with uACP could be partially explained by residual selection bias not addressed by our model. Also, the use of uACP and bACP may be a surrogate marker of surgeons and teams being more proactive and contemporary of several aspects of care, over and above cerebral protection, in the management of TAAD. Another important limitation of the present study was that the NACSA dataset is not designed specifically to capture information for patients undergoing surgery for TAAD and this may translate into inconsistent data entry. Some patients had incomplete information for the present analysis and were excluded. Finally, we had no information on core temperature but only whether deep versus moderate hypothermia was used, and no information about the extent of the dissection (i.e. involvement of carotid arteries or mesenteric vessels) and whether organ malperfusion was present at the presentation. For these reasons, our findings should be considered exploratory and hypothesis-generating rather than explanatory.

## CONCLUSION

In conclusion, the present analysis supports the hypothesis that for a short period of CA and in patients with less complex repair, uACP via the right subclavian artery (in isolation or combined with DH) can provide adequate cerebral perfusion while minimizing the risk related to direct carotid vessels manipulation. Conversely, bACP should be considered in patients requiring a longer period of CA and more complex vascular repair (i.e. arch replacement). The observed advantage from uACP may be partially attributed to the adoption of concomitant DH and the role of moderate hypothermia during uACP requires further investigation.

## SUPPLEMENTARY MATERIAL

Supplementary material is available at *EJCTS* online.

## Supplementary Material

ezab192_Supplementary_Data

## Notes

Presented at the 34th Annual Meeting of the European Association for Cardio-Thoracic Surgery, Barcelona, Spain, 8–10 October 2020.

## ABBREVIATIONS

ACP: Antegrade cerebral perfusion
bACP: Bilateral antegrade cerebral perfusion
CA: Circulatory arrest
CI: Confidence interval
CVA: Cerebrovascular accident
DH: Deep hypothermia
DHCA: Deep hypothermic circulatory arrest
NACSA: National Adult Cardiac Surgery Audit
OR: Odds ratio
RCP: Retrograde cerebral perfusion
SD: Standard deviation
TAAD: Type A aortic dissection
uACP: Unilateral antegrade cerebral perfusion
