# Supplementary material for: Neuroprotective strategies in acute aortic dissection: an analysis of the UK National Adult Cardiac Surgical Audit
Source: Eur J Cardiothorac Surg. 2021 May 8;60(6):1437–44. doi: 10.1093/ejcts/ezab192 (PMC8643475; doi:10.1093/ejcts/ezab192)
Supplement: ezab192_Supplementary_Data [file ezab192_supplementary_data.zip › Supplemental material_R1.docx]

SUPPLEMENTAL MATERIAL

Neuroprotective strategies in acute aortic dissection. An analysis of the UK National Adult Cardiac Surgical Audit.

Benedetto et al.

**CONTENTS**

**Supplementary Table I.** Incidence of primary and secondary endpoints across neuroprotective strategy groups stratified by duration of circulatory arrest.

**Supplementary Table II.** Incidence of primary and secondary endpoints across neuroprotective strategy groups without concomitant deep hypothermia stratified by duration of circulatory arrest.

**Supplementary Table III.** Incidence of primary and secondary endpoints across neuroprotective strategy groups with concomitant deep hypothermia stratified by duration of circulatory arrest.

**Supplementary Table IV.** Collinearity diagnostics of the generalized linear mixed-effect model presented in Table 3.

**Supplementary Table V.** Baseline characteristics of patients across group stratified by extension of surgical repair.

**Supplementary Table VI.** Incidence of primary and secondary endpoints across neuroprotective strategy groups stratified by extension of surgical repair.

**Supplementary Figure I.** Circulatory arrest time distribution with relative mean (x) and standard deviation (s) (50^th^ percentile: 30 min, 75^th^ percentile 45 min).

**Supplementary Table I.** Incidence of primary and secondary endpoints across neuroprotective strategy groups stratified by duration of circulatory arrest (CA)

|  | **CA <30** | | | | | | | **CA >=30** | | | | | | |
| --- | --- | --- | --- | --- | --- | --- | --- | --- | --- | --- | --- | --- | --- | --- |
|  | **DHCA only  (N=388)** | | **uACP (N=32)** | **bACP (N=236)** | | **RCP (N=100)** | | **DHCA only (N=290)** | | **uACP (N=46)** | | **bACP (N=384)** | | **RCP (N=100)** |
| **Death or CVA** |  |  | | |  | |  |  |  | |  | |  | |
| No | 285 (73.5%) | 28 (87.5%) | | | 170 (72.0%) | | 78 (78.0%) | 207 (71.4%) | 32 (69.6%) | | 256 (66.7%) | | 67 (67.0%) | |
| Yes | 103 (26.5%) | 4 (12.5%) | | | 66 (28.0%) | | 22 (22.0%) | 83 (28.6%) | 14 (30.4%) | | 128 (33.3%) | | 33 (33.0%) | |
| **Death** |  |  | | |  | |  |  |  | |  | |  | |
| No | 324 (83.5%) | 30 (93.8%) | | | 201 (85.2%) | | 85 (85.0%) | 238 (82.1%) | 35 (76.1%) | | 304 (79.2%) | | 78 (78.0%) | |
| Yes | 64 (16.5%) | 2 (6.2%) | | | 35 (14.8%) | | 15 (15.0%) | 52 (17.9%) | 11 (23.9%) | | 80 (20.8%) | | 22 (22.0%) | |
| **CVA** |  |  | | |  | |  |  |  | |  | |  | |
| No | 331 (85.3%) | 28 (87.5%) | | | 199 (84.3%) | | 89 (89.0%) | 246 (84.8%) | 43 (93.5%) | | 323 (84.1%) | | 84 (84.0%) | |
| Yes | 57 (14.7%) | 4 (12.5%) | | | 37 (15.7%) | | 11 (11.0%) | 44 (15.2%) | 3 (6.5%) | | 61 (15.9%) | | 16 (16.0%) | |

CA circulatory arrest time; CVA cerebrovascular accidents, uACP unilateral antegrade cerebral perfusion; bACP bilateral antegrade cerebral perfusion; RCP retrograde cerebral perfusion; DHCA deep hypothermic circulatory arrest.

**Supplementary Table II.** Incidence of primary and secondary endpoints across neuroprotective strategy groups without concomitant deep hypothermia stratified by duration of circulatory arrest.

|  | **CA <30 no DH** | | | **CA >=30 no DH** | | |
| --- | --- | --- | --- | --- | --- | --- |
|  | **uACP (N=14)** | **bACP (N=81)** | **RCP (N=22)** | **uACP (N=11)** | **bACP (N=127)** | **RCP (N=25)** |
| **Death or CVA** |  |  |  |  |  |  |
| No | 12 (85.7%) | 62 (76.5%) | 16 (72.7%) | 7 (63.6%) | 85 (66.9%) | 14 (56.0%) |
| Yes | 2 (14.3%) | 19 (23.5%) | 6 (27.3%) | 4 (36.4%) | 42 (33.1%) | 11 (44.0%) |
| **Death** |  |  |  |  |  |  |
| No | 12 (85.7%) | 71 (87.7%) | 20 (90.9%) | 9 (81.8%) | 95 (74.8%) | 17 (68.0%) |
| Yes | 2 (14.3%) | 10 (12.3%) | 2 (9.1%) | 2 (18.2%) | 32 (25.2%) | 8 (32.0%) |
| **CVA** |  |  |  |  |  |  |
| No | 12 (85.7%) | 68 (84.0%) | 18 (81.8%) | 9 (81.8%) | 110 (86.6%) | 20 (80.0%) |
| Yes | 2 (14.3%) | 13 (16.0%) | 4 (18.2%) | 2 (18.2%) | 17 (13.4%) | 5 (20.0%) |

CA circulatory arrest time; CVA cerebrovascular accidents, uACP unilateral antegrade cerebral perfusion; bACP bilateral antegrade cerebral perfusion; RCP retrograde cerebral perfusion; DHCA deep hypothermic circulatory arrest.

**Supplementary Table III.** Incidence of primary and secondary endpoints across neuroprotective strategy groups with concomitant deep hypothermia stratified by duration of circulatory arrest.

|  |  | **CA <30 DH** | | | |  | **CA >=30 DH** | | | |
| --- | --- | --- | --- | --- | --- | --- | --- | --- | --- | --- |
|  | **DHCA only (N=388)** | | **uACP (N=18)** | **bACP (N=160)** | **RCP (N=78)** | **DHCA only (N=290)** | | **uACP (N=35)** | **bACP (N=252)** | **RCP (N=75)** |
| **Death or CVA** |  | |  |  |  |  | |  |  |  |
| No | 285 (73.5%) | | 16 (88.9%) | 112 (70.0%) | 62 (79.5%) | 207 (71.4%) | | 25 (71.4%) | 167 (66.3%) | 53 (70.7%) |
| Yes | 103 (26.5%) | | 2 (11.1%) | 48 (30.0%) | 16 (20.5%) | 83 (28.6%) | | 10 (28.6%) | 85 (33.7%) | 22 (29.3%) |
| **Death** |  | |  |  |  |  | |  |  |  |
| No | 324 (83.5%) | | 18 (100%) | 135 (84.4%) | 65 (83.3%) | 238 (82.1%) | | 26 (74.3%) | 204 (81.0%) | 61 (81.3%) |
| Yes | 64 (16.5%) | | 0 (0%) | 25 (15.6%) | 13 (16.7%) | 52 (17.9%) | | 9 (25.7%) | 48 (19.0%) | 14 (18.7%) |
| **CVA** |  | |  |  |  |  | |  |  |  |
| No | 331 (85.3%) | | 16 (88.9%) | 135 (84.4%) | 71 (91.0%) | 246 (84.8%) | | 34 (97.1%) | 209 (82.9%) | 64 (85.3%) |
| Yes | 57 (14.7%) | | 2 (11.1%) | 25 (15.6%) | 7 (9.0%) | 44 (15.2%) | | 1 (2.9%) | 43 (17.1%) | 11 (14.7%) |

CA circulatory arrest time; CVA cerebrovascular accidents, uACP unilateral antegrade cerebral perfusion; bACP bilateral antegrade cerebral perfusion; RCP retrograde cerebral perfusion; DHCA deep hypothermic circulatory arrest.

**Supplementary Table IV.** Collinearity diagnostics of the generalized linear mixed-effect model presented in Table 3.

|  | **GVIF** |
| --- | --- |
| Age | 1.163429 |
| Female gender | 1.065243 |
| Known Marfan | 1.052272 |
| Critical preoperative state | 1.094898 |
| Emergency | 1.126397 |
| Neurological Dysfunction | 1.047778 |
| Diabetes | 1.015269 |
| Chronic pulmonary disease | 1.020214 |
| Extracardiac arteriopathy | 1.055642 |
| Previous cardiac surgery | 1.085353 |
| Creatinine > 200mmol/l | 1.05884 |
| Aortic Valve Surgery | 1.347533 |
| Aortic involvement | 1.659059 |
| CABG | 1.082614 |

GVIF generalized variance inflation factor; Df degree of freedom

**Supplementary Table V.** Baseline characteristics of patients across group stratified by extension of surgical repair.

|  | **Extent of surgical repair** | | | |
| --- | --- | --- | --- | --- |
|  | **Aortic root replacement  (N=444)** | **Aortic arch replacemet  (N=71)** | **Root and Arch replacement   (N=21)** | **Ascending aorta only (interposition graft) (N=1393)** |
| **Age (years)** |  |  |  |  |
| Mean (SD) | 56.3 (13.9) | 59.8 (13.1) | 58.8 (13.7) | 64.4 (12.8) |
| **Female gender** | 137 (30.9%) | 20 (28.2%) | 5 (23.8%) | 502 (36.0%) |
| **Known Marfan** | 34 (7.7%) | 4 (5.6%) | 1 (4.8%) | 26 (1.9%) |
| **Critical preoperative state** | 34 (7.7%) | 5 (7.0%) | 0 (0%) | 102 (7.3%) |
| **Emergency** | 389 (87.6%) | 43 (60.6%) | 11 (52.4%) | 1154 (82.8%) |
| **Neurological deficit** | 28 (6.3%) | 3 (4.2%) | 2 (9.5%) | 127 (9.1%) |
| **Diabetes** | 16 (3.6%) | 2 (2.8%) | 1 (4.8%) | 49 (3.5%) |
| **Chronic pulmonary disease** | 33 (7.4%) | 6 (8.5%) | 2 (9.5%) | 109 (7.8%) |
| **Extracardiac arteriopathy** | 70 (15.8%) | 27 (38.0%) | 7 (33.3%) | 295 (21.2%) |
| **Previous cardiac surgery** | 25 (5.6%) | 14 (19.7%) | 2 (9.5%) | 77 (5.5%) |
| **Creatinine >200mmol/l** | 18 (4.1%) | 4 (5.6%) | 0 (0%) | 51 (3.7%) |
| **Aortic valve surgery** | 340 (76.6%) | 10 (14.1%) | 15 (71.4%) | 318 (22.8%) |
| **Neuroprotective strategy** |  |  |  |  |
| uACP | 26 (5.9%) | 7 (9.9%) | 1 (4.8%) | 83 (6.0%) |
| bACP | 157 (35.4%) | 49 (69.0%) | 14 (66.7%) | 540 (38.8%) |
| DHCA only | 215 (48.4%) | 11 (15.5%) | 5 (23.8%) | 599 (43.0%) |
| RCP | 46 (10.4%) | 4 (5.6%) | 1 (4.8%) | 171 (12.3%) |
| **CABG** | 68 (15.3%) | 8 (11.3%) | 1 (4.8%) | 151 (10.8%) |
| **Cumulative cross clamp time (min)** |  |  |  |  |
| Mean (SD) | 178 (73.1) | 138 (66.4) | 230 (108) | 112 (55.3) |
| **Circulatory arrest time (min)** |  |  |  |  |
| Mean (SD) | 38.8 (34.1) | 54.0 (50.3) | 53.2 (29.0) | 35.8 (24.8) |

bACP bilateral antegrade cerebral perfusion; CABG coronary artery bypass grafting; DHCA deep hypothermic circulatory arrest; RCP retrograde cerebral perfusion; uACP unilateral antegrade cerebral perfusion.

**Supplementary Table VI.** Incidence of primary and secondary endpoints across neuroprotective strategy groups stratified by extension of surgical repair.

|  |  |  | | **Root** | | | **Arch** | | | | **Root and Arch** | | |  | | **Ascending aorta** | | | |  |
| --- | --- | --- | --- | --- | --- | --- | --- | --- | --- | --- | --- | --- | --- | --- | --- | --- | --- | --- | --- | --- |
|  | **DHCA (N=215)** | | **uACP (N=26)** | | **bACP (N=157)** | **RCP (N=46)** | **DHCA (N=11)** | **uACP (N=7)** | **bACP (N=49)** | **RCP (N=4)** | **DHCA (N=5)** | **uACP (N=1)** | **bACP (N=14)** | | **RCP (N=1)** | **DHCA (N=599)** | **uACP (N=83)** | **bACP (N=540)** | **RCP (N=171)** | |
| **Death or CVA** |  | |  | |  |  |  |  |  |  |  |  |  | |  |  |  |  |  | |
| No | 165 (76.7%) | | 20 (76.9%) | | 104 (66.2%) | 33 (71.7%) | 6 (54.5%) | 6 (85.7%) | 34 (69.4%) | 2 (50.0%) | 4 (80.0%) | 1 (100%) | 9 (64.3%) | | 0 (0%) | 431 (72.0%) | 64 (77.1%) | 390 (72.2%) | 125 (73.1%) | |
| Yes | 50 (23.3%) | | 6 (23.1%) | | 53 (33.8%) | 13 (28.3%) | 5 (45.5%) | 1 (14.3%) | 15 (30.6%) | 2 (50.0%) | 1 (20.0%) | 0 (0%) | 5 (35.7%) | | 1 (100%) | 168 (28.0%) | 19 (22.9%) | 150 (27.8%) | 46 (26.9%) | |
| **Death** |  | |  | |  |  |  |  |  |  |  |  |  | |  |  |  |  |  | |
| No | 186 (86.5%) | | 20 (76.9%) | | 120 (76.4%) | 37 (80.4%) | 8 (72.7%) | 6 (85.7%) | 44 (89.8%) | 3 (75.0%) | 5 (100%) | 1 (100%) | 10 (71.4%) | | 0 (0%) | 493 (82.3%) | 71 (85.5%) | 451 (83.5%) | 139 (81.3%) | |
| Yes | 29 (13.5%) | | 6 (23.1%) | | 37 (23.6%) | 9 (19.6%) | 3 (27.3%) | 1 (14.3%) | 5 (10.2%) | 1 (25.0%) | 0 (0%) | 0 (0%) | 4 (28.6%) | | 1 (100%) | 106 (17.7%) | 12 (14.5%) | 89 (16.5%) | 32 (18.7%) | |
| **CVA** |  | |  | |  |  |  |  |  |  |  |  |  | |  |  |  |  |  | |
| No | 187 (87.0%) | | 24 (92.3%) | | 138 (87.9%) | 39 (84.8%) | 9 (81.8%) | 7 (100%) | 37 (75.5%) | 3 (75.0%) | 4 (80.0%) | 1 (100%) | 12 (85.7%) | | 1 (100%) | 512 (85.5%) | 74 (89.2%) | 462 (85.6%) | 150 (87.7%) | |
| Yes | 28 (13.0%) | | 2 (7.7%) | | 19 (12.1%) | 7 (15.2%) | 2 (18.2%) | 0 (0%) | 12 (24.5%) | 1 (25.0%) | 1 (20.0%) | 0 (0%) | 2 (14.3%) | | 0 (0%) | 87 (14.5%) | 9 (10.8%) | 78 (14.4%) | 21 (12.3%) | |

CVA cerebrovascular accidents, uACP unilateral antegrade cerebral perfusion; bACP bilateral antegrade cerebral perfusion; RCP retrograde cerebral perfusion; DHCA deep hypothermic circulatory arrest.
