# Supplementary figures and images for: Neuroprotective strategies in acute aortic dissection: an analysis of the UK National Adult Cardiac Surgical Audit
Source: Eur J Cardiothorac Surg. 2021 May 8;60(6):1437–44. doi: 10.1093/ejcts/ezab192 (PMC8643475; doi:10.1093/ejcts/ezab192)

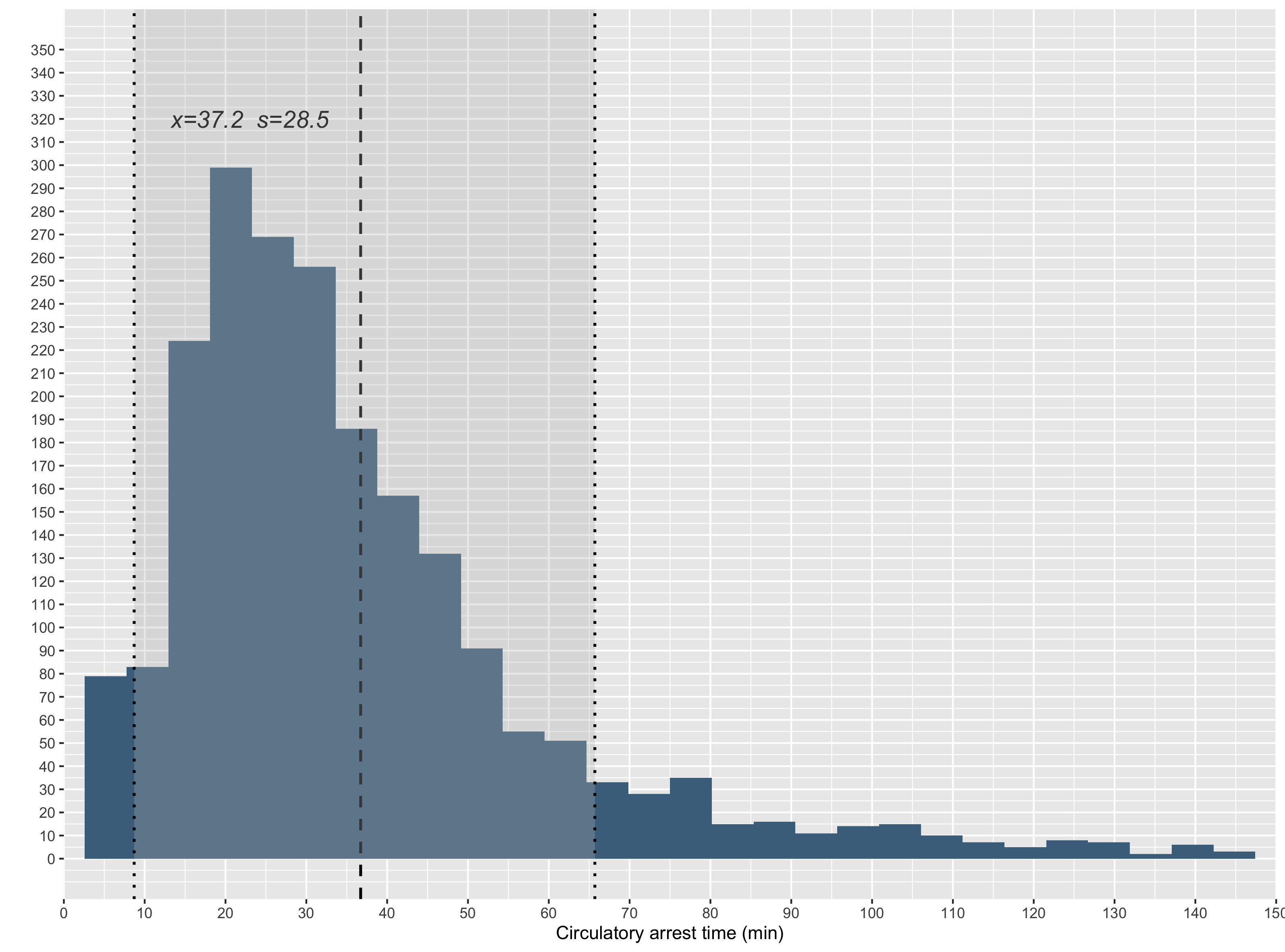

Supplement: ezab192_Supplementary_Data [file ezab192_supplementary_data.zip › Suppl.Fig 1.tiff]
